# An Engineered Floating Nanoparticulate System to Modulate Biopharmaceutical Performance and Therapeutic Efficacy of Tapentadol in Chronic Neuropathic Pain Management

**DOI:** 10.3390/pharmaceutics18101282

**Published:** 2026-10-10

**Authors:** Ranju Soni, Sheelu Sharma, Manu Sharma

**Affiliations:** 1 Department of Pharmacy, Banasthali Vidyapith, Rajasthan 304022, India; 2 Pandit Deendayal Upadhyaya, National Institute for Persons with Disabilities (Divyangjan) (PDUNIPPD), Delhi University, New Delhi 110002, India

**Keywords:** buoyancy, neuropathic pain, tapentadol, floating nanoparticles, bioavailability

## Abstract

**Background:** Tapentadol is an effective centrally acting analgesic for treating neuropathic pain. However, its short half-life, high first-pass metabolism, and requirement for frequent dosing limit its therapeutic effect and patient compliance. The purpose of the present innovation was to develop cost-effective tapentadol-loaded floating nanoparticles (TFNs) for sustained oral delivery. **Methods:** TFNs were prepared using the double emulsion solvent evaporation method. The effects of various formulation and process parameters, including polymer ratio, drug concentration, surfactant concentration and type, sonication time, and external phase volume were systematically studied. Analytical physicochemical characterization, morphology, buoyancy and drug release behavior were carried out. In vivo performance was evaluated by performing pharmacokinetic, tissue distribution, and pharmacodynamics studies in a chronic constriction injury model. **Results:** The optimized formulation exhibited spherical shape with nano-size (320.66 ± 17.50 nm), and good entrapment efficiency (82.85 ± 6.57%). Analytical characterization confirmed successful drug encapsulation and compatibility with formulation components. The engineered floating nanoparticulate architecture allowed both long gastric drug retention and a controlled drug release, governed by an anomalous diffusion-controlled mechanism. Pharmacokinetic evaluation revealed 3.36-fold higher relative oral bioavailability from TFNs. In addition, 1.49-fold higher drug availability in brain with TFNs was observed. Pharmacodynamic studies demonstrated a marked reduction in neuropathic pain responses, confirming the superior and prolonged analgesic effect of TFNs. **Conclusions:** The findings establish the developed TFNs as a promising controlled-release system for effective and sustained management of neuropathic pain. Even so, further studies focusing on large-scale production, long-term safety, and clinical evaluation are necessary before the formulation can be advanced for clinical application.

## 1. Introduction

Chronic neuropathic pain, emerging continuously or in episodes, is a major debilitating healthcare condition. It may arise due to damage or dysfunction of the somatosensory nervous system, which leads to complex alterations in cognitive and emotional brain functions of the sufferer [1,2]. The available treatments are often refractory due to its complex pathophysiology encompassing anomalous nociceptive signaling, central sensitization and neuroplastic changes [3]. Anticonvulsants; antidepressants; and local anesthetics, opioids, and non-steroidal anti-inflammatory drugs (NSAIDS) are among the treatment choices clinically prescribed for neuropathic pain management [4]. However, the benefits of long-term therapy are limited by the development of tolerance and the danger of related side effects. The effectiveness of customized combination regimens of multimodal analgesia in individuals has been consistently demonstrated by randomized clinical trials. However, tolerance development and concomitant drug–drug interactions can complicate chronic neuropathic pain management [5]. Therefore, the use of a multimodal analgesic with long-term safety capable of enhancing a person’s working ability is needed for efficient neuropathic pain management.

Tapentadol (TAP), a FDA-approved, centrally acting synthetic opioid analgesic, works as an agonist at the µ-opioid receptor and noradrenaline reuptake inhibitor. Thus, TAP’s dual synergistic mode of action allows it to reduce a wide range of nociceptive and neuropathic pain, as well as the risk of any unwanted drug–drug interaction [6,7,8]. In addition, it is better tolerated than other opioids such as hydrocodone, oxycodone, and morphine [9]. It is quickly absorbed from its widely available conventional tablets. However, its exceedingly bitter taste, high first-pass metabolism contributing to poor bioavailability (32%) and shorter half-life (4 h), coupled with repeated dosing, ultimately result in non-adherence to its therapy. In addition, TAP is known to exhibit higher inter-subject variability [9]. Tapentadol (TAP), marketed as NUCYNTA^®^, is the only FDA-approved extended-release formulation currently indicated for the management of chronic osteoarthritis, low back pain, and pain stemming from diabetic peripheral neuropathy. However, NUCYNTA^®^ therapeutic efficacy depends upon its intact ingestion. Chewing, crushing, or dissolving such formulations can lead to dose dumping, resulting in potentially life-threatening toxicity and overdose [10]. In addition, TAP’s primary absorption site lies in the upper gastrointestinal tract, especially the stomach and proximal small intestine, making prolonged gastric residence essential to maximize systemic absorption and bioavailability [11,12]. Therefore, there is a critical need to develop a novel controlled-release formulation that addresses these challenges while improving patient adherence.

Gastroretentive technology thus offers a formulation approach to achieve extended pharmacokinetic response from a traditional rapid drug input, potentially enhancing the therapeutic response and patient convenience. Among the various gastroretentive strategies, floating systems have a relatively simple and non-invasive mechanism for achieving gastroretention compared to mucoadhesive systems, which interact with the continually renewed gastric mucus layer, or expandable and high-density systems, which necessitate a certain dimensional and/or density property to prevent gastric emptying [13,14]. The floating platform can be formulated as a hydrophilic polymeric matrix, where the gastric residence is maintained by buoyancy and the release can be controlled by hydration, gel formation and matrix erosion/diffusion [14,15], although functionality of conventional floating tablets is inherently dependent on tablet properties, integrity of the tablet matrix, hydration/ swelling effect and drug release rate from the tablet [16]. Single-unit gastroretentive systems can have a variable gastric retention time, as the early release of the single unit may lead to the loss of the desired drug release profile. Multiparticulate systems are therefore of interest as they offers more consistent drug release profiles [17,18]. In addition, multiparticulate systems can potentially circumvent the susceptibility of dose dumping or premature gastric expulsion [17].

The feasibility of gastroretentive delivery of tapentadol has previously been demonstrated using conventional floating tablets capable of sustained drug release, together with prolonged in vitro buoyancy [19]. Nanoparticulate platforms have also been investigated for the administration of tapentadol, such as chitosan nanoparticles for intranasal administration and other nanoparticulate platforms mainly focusing on the modification of drug release or drug absorption [20,21]. Furthermore, clinically available prolonged-release tapentadol already has a delayed time to peak concentration, with prolonged exposure compared to immediate-release tapentadol, demonstrating that modulation of the rate of systemic drug input can be pharmacokinetically meaningful [22]. Therefore, the formulation challenge of achieving a longer unmet need for tapentadol is not just one of prolonging the drug release, as has been achieved by conventional prolonged release, floating and nanoparticulate systems, but to develop and demonstrate a multifunctional oral system, in which nanodispersed drug release, gastric buoyancy and sustained release are all combined to dynamically control the systemic input of the drug, and ultimately its therapeutic activity in neuropathic pain management.

In the selection of an appropriate polymer, its concentration, viscosity grade, molecular weight, swelling capacity, and gel strength are key parameters influencing the performance of such a system, since the polymer influences the release rate, hydration, swelling, matrix formation, particle integrity, buoyancy and drug-diffusion properties [18]. Hydrophilic polymers like hydroxypropyl methylcellulose (HPMC) variants (K4M, K15M, K100M), hydroxyl propyl cellulose, chitosan, Carbopol, polyethylene oxide, etc., can be employed individually or in combination to tailor the release kinetics, floating duration, and mechanical properties of the formulation [23].

Considering the above-discussed facts, we developed TAP-loaded floating nanoparticles to enhance gastric residence time. The rationale behind the TFN formulation development was to prolong the time for TAP to be released, and then its availability in the GI tract for absorption at downstream absorptive sites, such as the intestine. Therefore, gastric retention was viewed as a “controlled drug-input” strategy which aimed to slow down the rapid gastrointestinal transit without relying on preferential gastric absorption and, hence, providing a sustained supply of TAP for absorption. Polymer combination and processing variables were optimized to establish balance among polymer hydration, swelling, buoyancy and diffusion resistance to enable prolonged gastric residence and sustained tapentadol release. Pharmacokinetic, bio-distribution and pharmacodynamic investigations were performed to evaluate the potential of TFNs in enhancing bioavailability and therapeutic benefits.

## 2. Materials and Methods

### 2.1. Chemicals

Tapentadol (TAP) was received as a complimentary sample from MSN Laboratories Pvt. Ltd. (Hyderabad, India). Ethocel (EC) and methocel (MC) were purchased from Merck, Germany. Poly (vinyl alcohol) (PVA), acetonitrile (ACN,) and HPLC water were procured from Merck (Mumbai, India). Tween 80, Span 80, ethanol, isopropyl alcohol and dichloromethane were procured from CDH, India. All experiments were performed using standard laboratory-grade chemicals and distilled water.

### 2.2. Preparation of TFNs

Tapentadol-loaded floating nanoparticles (TFNs) were prepared via double emulsion solvent evaporation method [24]. Different weight ratios of Ethocel (EC) and Methocel (MC) were dissolved in a solvent, i.e., blend of ethanol, dichloromethane and isopropyl alcohol in a 3:2.5:2 ratio containing Span 80 (0.5% *w*/*v*) as a surfactant. TAP aqueous solution was added dropwise in above solution and probe sonicated at 40 W, 60% amplitude for 10.5 min. Milky white primary emulsion so formed was gradually added dropwise into PVA solution (1% *w*/*v*, 30 mL) being probe sonicated at 40 W, 60% amplitude to prepare double emulsion (W_1_/O/W_2_). The emulsion was agitated at 1200 rpm for 16 h under ambient conditions to facilitate complete removal of solvents. The nanoparticles were subsequently isolated by centrifugation at 20,000 rpm for 25 min at 4 °C. Supernatant collected was evaluated for free drug content, as described later. Pellet of nanoparticles produced was then dispersed in distilled water containing 10% *w*/*v* mannitol and freeze dried for 36 h at −80 °C and 0.05 mbar pressure. The sequential one-variable-at-a-time (OVAT) method was used for formulation optimization. The EC:MC polymer ratio (2:1 to 1:2), the drug:polymer (1:2.67 to 1.8) ratio, the type and concentration (0.5 to 1.0% *w*/*v*) of the surfactant, pre-sonication time (7.5 to 12.5 min), post sonication time (10.5 to 15.0 min) and the external-phase volume (20 to 40 mL) were studied systematically. One formulation or process variable was manipulated for each experimental series, with all other variables held constant at the levels previously chosen. The impact of each variable on TFN critical quality attributes such as particle size, PDI, zeta potential and entrapment efficiency was determined. The optimized value for each variable was based on the target range set for these critical variables, and the next variable was subsequently optimized. All experiments were conducted in triplicate, and results are reported as mean ± standard deviation to reflect response variability.

### 2.3. Characterization of TFNs

#### 2.3.1. Particle Size and Zeta Potential

Particle size, polydispersity index (PDI), and zeta potential of TFNs were determined using photon correlation spectroscopy with a Nano ZS Malvern Zetasizer (Malvern Panalytical, Malvern, Worcestershire, UK) ). Before measurement, each lyophilized sample was dispersed and sufficiently diluted (1:100 *v*/*v*) with distilled water. Subsequently, dispersions were allowed to equilibrate at 25 ± 0.5 °C for about 5 min prior to measurement. Three measurement runs for each sample were taken to determine the particle size, PDI and zeta potential. Each value represented was the mean of three independent replicates.

#### 2.3.2. Drug Entrapment

Entrapment efficacy of TFNs was evaluated by centrifuging the nanoparticle suspension at 20,000 rpm for 25 min at 4 °C. The resultant supernatant was collected and analyzed for free drug at 271 nm using a UV/Vis spectrophotometer (UV/Vis 3000+, Labindia, India). Blank formulation (containing all excipients, polymers, PVA and surfactant without TAP) samples were analyzed with the same sample processing procedure as the nanoparticle samples. The absorbance of the formulation blank was recorded at 271 nm, which was then compared with the absorbance of the TAP standards. TAP calibration curve was prepared in the range of concentrations 1–10 µg/mL, and the linearity was assessed by regression analysis (R^2^ = 0.99). No significant absorbance contribution from the blank formulation at 271 nm was deemed to indicate minimal interference caused by the formulation components. The percentage entrapment of TAP was determined using the following expression:

(1) $$\mathrm{Entrapment}\;\mathrm{efficiency}\;\left(\%\right)=\frac{\left(\mathrm{Total}\;\mathrm{drug}\;\mathrm{added}-\mathrm{Free}\;\mathrm{drug}\;\mathrm{in}\;\mathrm{supernatant}\right)}{\mathrm{Total}\;\mathrm{drug}\;\mathrm{added}}\times 100$$

#### 2.3.3. Yield

Lyophilized TFNs were collected and weighed. The percentage yield was determined by following formula:

(2) $$\mathrm{Yield}\;\left(\%\right)=\frac{\mathrm{Actual}\;\mathrm{weight}\;\mathrm{of}\;\mathrm{product}}{\mathrm{Total}\;\mathrm{weight}\;\mathrm{of}\;\mathrm{drug}\;\mathrm{and}\;\mathrm{excipients}}\times 100$$

#### 2.3.4. Buoyancy and Swelling Index

Concisely, TFNs (200 mg) were gently sprinkled over simulated gastric fluid (SGF pH 1.2) under continuous stirring at 50 rpm. The nanoparticles were observed over a period of 12 h to monitor which fractions remained floating and settled at the bottom of SGF. Later, floating and settled nanoparticles were carefully collected separately. Both fractions were then dried to remove any residual moisture and accurately weighed [17]. The buoyancy was estimated using the formula:

(3) $$\mathrm{Buoyancy}(\%)=\;\frac{\mathrm{QF}}{\mathrm{QF}+\mathrm{QS}}\times 100$$

where *QF* and *QS* are the weights of the floating and the settled nanoparticles, respectively.

Simultaneously, the particle size enlargement was measured over a period of 12 h by measuring the weight of the swollen nanoparticles collected after filtration. Excess buffer adhering to nanoparticles was carefully blotted off using tissue paper to avoid inaccuracies in weight measurement [17]. The swelling behavior of the optimized formulation was determined utilizing the following equation:

(4) $$\mathrm{Swelling}\;\mathrm{index}\;\left(\%\right)=\frac{\mathrm{wt}-w}{w}\times 100$$

where ‘*wt*’ represents the weight of the swollen nanoparticles at a given time interval, and ‘*w*’ represents the initial weight of the nanoparticles.

#### 2.3.5. ATR-FTIR Spectral Analysis

ATR-FTIR spectra of tapentadol, EC, MC, and the optimized formulation were recorded using a Bruker EQUINOX 55 FTIR spectrophotometer between 4000 and 600 cm^−1^ at a resolution of 2 cm^−1^. An advanced ATR correction was applied to all spectra before recording the percentage transmittance.

#### 2.3.6. Differential Scanning Calorimetry (DSC)

Thermal characteristics of tapentadol, EC, MC, their physical mixture, and the optimized TFNs were evaluated utilizing NETEZCH DSC 204 F1 phoenix. Samples packed in aluminum pans were heated from 30 to 250 °C at a constant heating rate of 10 °C per min. The resulting thermograms were evaluated for thermal behavior and possible interaction with the formulation component.

#### 2.3.7. Powder X-Ray Diffraction (PXRD)

XPERT-PRO X-ray diffractometer fitted with CuKα radiation (45 kV, 40 mA) was utilized to evaluate diffraction patterns between 5° and 50° 2θ of tapentadol, EC, MC, their physical mixture, and optimized TFNs. The recorded diffractograms were analyzed to evaluate the crystallinity of the drug and identify any change in crystallinity following nanoparticle formulation.

#### 2.3.8. Scanning Electron Microscopy (SEM)

Prior to analysis, lyophilized nanoparticles were evenly distributed on double-sided carbon adhesive tape mounted on aluminum specimen stub. Before SEM examination, a fine coating of gold was applied to sample under high-vacuum conditions using a Polaron coating system to improve electrical conductivity and facilitate high quality. The coated samples were subsequently observed under SEM, and the surface characteristics and particle morphology were recorded at suitable magnification.

### 2.4. In Vitro Release

Drug release from pure drug and optimized TFNs was determined using the dialysis membrane technique. Optimized TFNs, corresponding to 3 mg TAP placed into dialysis bag (molecular cut off 6000–8000 Da) was immersed in dissolution medium (SGF pH 1.2, 100 mL) kept at 37 ± 0.5 °C with continuous stirring at 100 rpm. At predestined durations (0.5, 1, 2, 4, 6, 8, 10, 12 and 24 h), samples (3 mL) were withdrawn from dissolution medium, filtered and examined by a UV/Visible spectrophotometer. After each sample withdrawn from the dissolution medium, an equal amount of fresh medium (3 mL) was added to keep the total amount unchanged and ensure sink conditions. The volume of release medium (100 mL) was sufficient to maintain sink conditions as TAP having high saturation solubility (~34 g/100 mL), which is significantly greater than the concentration achieved during release study. The amount of tapentadol released at each time point was measured at 271 nm. The release data were analyzed with distinct kinetic mathematical models like zero-order, first-order, Higuchi, and Korsmeyer–Peppas models to evaluate the drug release mechanism.

### 2.5. Stability Studies

Stability of optimized lyophilized TFNs was estimated according to ICH guidelines for climatic zones III and IV at both accelerated (40 ± 2 °C and 75 ± 5% RH) and room temperature (25 ± 2 °C and 60 ± 5% RH) storage stability conditions over a period of 6 months. Each sample was analyzed for various parameters, including particle size, PDI, zeta potential, and in vitro drug release behavior. Additionally, the visual appearance of the formulation was inspected to detect any changes in color or other physical properties.

### 2.6. In Vivo Studies

Wistar rats weighing 200 ± 20 g were obtained from the institutional animal house and maintained under standard laboratory conditions of temperature (22 ± 2 °C), humidity (55 ± 5%) and 12 h light/dark phase. The animals were provided with a standard laboratory diet and water ad libitum. All in vivo experiments were approved by the Institutional Animal Ethical Committee of Banasthali Vidyapith (574/GO/ReBi/S/02/CPCSEA), Rajasthan, India, and conducted in accordance with CPCSEA guidelines for the care and use of laboratory animals.

#### 2.6.1. Pharmacokinetic Evaluation

Overnight fasted Wistar rats maintained with water ad libitum were randomly categorized into two groups, each containing six rats. Group I received pure TAP solution, whereas Group II received the optimized TFNs at a dose providing 3 mg/kg of TAP. Blood samples (250 µL) were withdrawn from the rat tail vein at designated time spans of 0.5, 1, 2, 4, 6, 8, 12 and 24 h after administration. To maintain blood volume, an equal amount of sterile normal saline was administered after each blood collection. Blood samples were centrifuged to obtain plasma and analyzed for TAP concentration using reverse-phase high-performance liquid chromatography (LC-2010CHT, Shimadzu, Japan) equipped with a UV–Visible detector (Shimadzu Co., Kyoto, Japan) and a C18 analytical column (Phenomenex, Phenomenex Inc., CA, USA, 250 × 4.6 mm, 5 µm). Plasma samples were mixed with 0.4 mL of extraction solvent (ethanol: dichloromethane in 70:30, *v*/*v*) for 5 min. Subsequently, samples were centrifuged at 5000 rpm for 5 min. Organic phase was carefully transferred to clean glass tube and evaporated to dryness at 50 °C. The obtained residue was reconstituted in 70 μL mobile phase, vortexed for 1 min, and 20 μL of this solution was injected into the HPLC system. The separation was performed by chromatographic system with a mobile phase of 0.1% formic acid and acetonitrile (80:20 *v*/*v*) at 1.0 mL/min flow rate at 271 nm UV detection [11]. Each processed plasma sample was analyzed in triplicate, and the mean concentration was used for pharmacokinetic analysis. It was quantified by preparing an external calibration curve in the range of 50–10,000 ng/mL, which showed good linearity (R^2^ = 0.99). The extraction recovery rates for TAP were 80.86%, 85.69% and 83.63% at low, medium and high quality control concentrations, respectively. The analytical method showed a high accuracy of 98.76%, and the precision showed a relatively small coefficient of variation (%CV, 8.5%). The limit of detection (LOD) and lower limit of quantification (LLOQ) were 35 ng/mL and 50 ng/mL, respectively. The method was then used to determine the plasma concentration–time profiles of TAP after oral administration of TAP in pure form and TFNs. Non-compartmental analysis of the plasma concentration–time profiles of TAP after administration of pure TAP and TFNs was performed using GraphPad Prism version 8.2.2 (GraphPad Software, San Diego, CA, USA).

#### 2.6.2. Tissue Distribution Study

Healthy Wistar rats were assigned to two study groups with 12 rats in each group. Group I received an oral dose of TAP solution, whereas Group II received optimized TFNs corresponding to 3 mg/kg TAP. At selected time intervals of 1, 2, 4 and 8 h after dosing, 3 animals from each group were euthanized. The liver, brain and stomach were carefully excised, rinsed, and weighed. The tissues were then minced and homogenized. The homogenates were centrifuged at 12,000 rpm for 20 min. The resulting supernatants were collected to determine drug concentration [11].

#### 2.6.3. Pharmacodynamics Study

##### Chronic Constriction Injury (CCI) Model Development

Peripheral neuropathic pain was induced in healthy Wister rats using CCI model. Initially, the hair over the right thigh was carefully removed, and the surgical site was disinfected with Betadine™ solution (G. S. Pharmbutor Pvt. Ltd., India). Subsequently, animals were anesthetized and a small lateral surface of thigh skin was incised to reveal sciatic nerve of right hind limb. Sciatic nerve, located proximally to its trifurcation, was carefully freed from surrounding connective tissue. Four loose ligatures of black silk suture (4.0) were tied around the nerve at approximately 1.5 mm distance, ensuring that epineural circulation remained intact. Following ligation, the muscle and skin layers were sutured separately and Betadine™ applied to incision site to prevent risk of infection. Animals were placed in individual cages and allowed to recover. Sham-operated animals subjected to identical surgical intervention, including exposure of the sciatic nerve but no ligation or nerve injury performed [11,25]. Treatments specific to the experimental protocol were initiated on the day after surgery. Animals were allocated into four groups (n = 6). Group I served as vehicle control group and received normal saline (vehicle); Group II and Group III received tapentadol (3 mg/kg) and optimized formulation equivalent to 3 mg/kg tapentadol orally, respectively. Sham control group animals received normal saline (Group IV). Each treatment was administered based on the body weight of rats at 9:00 am daily, starting from the day of surgery to 21st day.

##### Rota Rod Test

Motor coordination and balancing were evaluated using Rota rod test following the administration of drug and formulation on days 3, 7, 14 and 21 [26,27]. To familiarize the rats with apparatus and minimize stress during testing, all rats underwent a training session on the Rota rod a day before the actual testing day. On the test day, rats were placed individually on a rotating rod set at a speed of 4 rpm. The rats were required to walk continuously against the motion of rods to maintain their balance. Each rat was gently placed on the rotating rod and the latency to fall was recorded as a measure of motor coordination and balance.

##### Von Frey Filament Test

Mechanical allodynia was quantified by estimating the paw withdrawal threshold triggered by mechanical stimulation using von Frey filaments (Model 2390; IITC Life Science Inc., Woodland Hills, CA, USA.). Each rat was placed individually in a clear plastic box (25 × 10 × 14 cm) with wire mesh bottom for 20 min to adapt this chamber prior to test. Von Frey filament (0.5 mm) was applied vertically to mid planter surface of same hind paw. This applied force was gradually increased from 1 to 50 g (at 1 g increments for 25 s) and held at the largest force (50 g) for 10 s if there was no withdrawal response. The paw withdrawal threshold (PWT) was used to determine the minimum force needed to cause withdrawal. All measures were repeated three times, with a 2 min time between each successive stimulation and a mean value recorded. Mechanical hypersensitivity was evaluated on days 3, 7, 14 and 21 after treatment [11,28].

### 2.7. Statistical Evaluation

Data were expressed as mean ± standard deviation (SD) where not otherwise indicated. Statistical analysis was performed using GraphPad Prism version 8.2.2 (GraphPad Software, San Diego, CA, USA). One-way analysis of variance (ANOVA) with Tukey multiple range test was used for comparison between the formulations prepared with different formulation and process variables. The biological replicates per experiment were reported separately for each experiment. The six animals in each treatment group were included in the pharmacokinetic study (n = 6/group), and the pharmacokinetic parameters were determined from the individual animal concentration–time profiles. Pharmacokinetic parameters were compared between groups with the use of one-way ANOVA followed by Tukey’s multiple comparisons test for post hoc comparisons. Three animals per treatment group per time point of interest (n = 3/group/time point) were used for the tissue distribution study. The two-way ANOVA, followed by Tukey’s multiple comparisons test for post hoc comparisons, was used to analyze the concentrations of tissue TAP. The number of animals per treatment group in each of the pharmacodynamic experiments was 6 (n = 6/group). Since the same animals were measured across all experimental days, the data were analyzed using repeated-measures two-way ANOVA, followed by Tukey’s multiple-comparisons test with treatment and time as fixed factors, and animal identity as the repeated-measures/subject factor. A *p* value < 0.05 was considered statistically significant.

## 3. Results and Discussion

### 3.1. Formulation Optimization

Various formulation parameters were optimized to develop TFNs with desirable characteristics, including nano-size range, high entrapment efficiency, and good colloidal stability. Methocel and Ethocel were utilized as key polymers to enhance the gastroretentive properties of the formulation.

EC:MC ratio played a crucial role in determining colloidal properties of nanoparticles for hydrophilic drug tapentadol [29]. A higher proportion of EC produced larger particles and poor entrapment efficiency due to its hydrophobic nature, though it provided a rigid structure. Table 1 represents that average particle size of different formulations varied from 320.66 ± 17.50 nm to 740.66 ± 53.68 nm. Decrease in mean particle diameter with increase in entrapment efficiency was obtained on alteration of EC:MC ratio from 2:1 to 1:1 (*p* < 0.05). Increase in MC significantly improved drug entrapment because of its hydrophilic and swelling characteristics (*p* < 0.05) [30]. However, increase in particle size was found with further increase in MC fraction in polymer amount (*p* < 0.05). This could be attributed to increased viscosity of polymer solutions, attributing to raised interfacial tension resulting in genesis of coarse dispersion [31]. In addition, a significant drop in entrapment efficiency was observed as MC fraction was increased in polymer amount (*p* < 0.05) (Table 1). This could be due to leaching out of TAP, a hydrophilic drug across the hydrophilic matrix formed by MC in TFNs. Among the various polymer ratios, 1:1 ratio of EC: MC exhibited the most favorable colloidal properties, including smallest particle size (320.66 ± 17.50 nm), low polydispersity index (0.12 ± 0.02), high zeta potential (−22.75 ± 2.68 mV), and maximum entrapment efficiency (82.85 ± 6.57%). These findings indicated that a balanced polymer ratio, EC:MC in 1:1 ratio produced stable nanoparticles with high drug loading and suitable colloidal characteristics for gastroretentive delivery [32,33].

A notable change in entrapment efficiency with insignificant change in particle size was observed when drug–polymer ratio was altered from 1:2.67 to 1:4 (*p* < 0.05). This might be attributed by the relatively higher polymer amount in solution, allowing quicker creation of film around core material, and its solidification preventing drug leaching by slowing down the solvents’ counter diffusion [34]. However, increasing the drug–polymer ratio from 1:4 to 1:8 did not produce a considerable increase in entrapment efficacy (Table 1). This might be due to lower amount of drug available for incorporation into nanoparticles.

Sonication time during both primary and secondary emulsification significantly affected particle size and drug entrapment efficiency (*p* < 0.05) (Table 1). On increasing the primary emulsification sonication time from 7.5 to 10.5 min produced a considerable decrease in particle size and an improvement in entrapment efficiency (*p* < 0.05), possibly due to the formation of a more uniform micellar structure. On the contrary, further increase in sonication time (12.5 min) contributed to elevation in particle size and reduction in encapsulation efficiency (*p* < 0.05) (Table 1). Elevated shear force experienced by system might have destabilized the system thermodynamically by distorting the interfacial surfactant barrier layer between two phases during micellization and enhanced drug leakage from the polymeric matrix [35]. Similarly, elevation of sonication time from 10.5 min to 12.5 min during secondary emulsification reduced particle size and increased encapsulation efficiency (*p* < 0.05) (Table 1). However, extending the sonication time to 15 min led to notable increase in particle size and reduced encapsulation efficiency by disturbing thermodynamic stability of the system (*p* < 0.05) [36].

During secondary emulsification, the interplay of shear forces and external phase volume significantly influenced vesicle characteristics. At low external phase volume (20 mL), excess shear disrupted vesicles to smaller sizes, leading to drug leakage and reduced entrapment efficiency (*p* < 0.05). On the contrary, a large external phase volume (40 mL) might have experienced low shear and yielded large colloidal particles, increased system heterogeneity, and lower entrapment efficiency (*p* < 0.05) [37].

The effect of emulsifier on TFN particle size was estimated while holding all other formulation variables fixed. Among the tested surfactants, Span 80 resulted in smaller-sized particles with high entrapment efficiency than Tween 80 (*p* < 0.05) (Table 1). The better performance of Span 80 can be linked to its lower HLB value, which allows it to form a stronger film around the particles and limits the escape of the hydrophilic drug into the outer aqueous phase. Previous reports have also shown that low-HLB surfactants such as Span 80 give higher stability and entrapment efficiency than more hydrophilic surfactants like Tween 80 [37,38]. Thus, Span 80 was identified as the more suitable surfactant for the formulation of TFNs. Surfactant concentration had pronounced effect on particle size. Increase in Span-80 concentration (1% *w*/*v*) had more pronounced effect on reduction in particle size, but reduced entrapment efficiency (*p* < 0.05). The particle size reduction in TFNs at higher surfactant concentration indicated that surfactant altered the interfacial area during formation of primary emulsion, but enhanced leakiness of vesicles formed [39,40].

The polydispersity index of batches varied from 0.11 to 0.95. Formulation batches with PDI ≤ 0.3 confirmed the homogeneity of colloidal system [41]. However, the stability of a colloidal system is mainly influenced by its surface charge, which is expressed as zeta potential. Zeta potential values for different formulations ranged from −9.63 to −24.86 mV. Formulations having zeta potential with numerical values near ±30 mV ensured considerable physical stability by preventing their coalescence or aggregation [41,42]. The observed zeta potential also showed that the surfactant concentration was sufficient to decrease the interfacial tension between the phases and facilitate stable micelle formation.

Among the prepared formulations, batch G2 exhibited the highest encapsulation efficiency (82.85 ± 6.57%) with particle size within nano-size range (320.66 ± 17.50 nm), higher zeta potential (−22.75 ± 2.68 mV) and a favorable PDI value (0.12 ± 0.02), indicating good colloidal stability. Therefore, batch G2 was selected as the optimized formulation for further investigations.

### 3.2. Swelling and Buoyancy

**Figure 1:**
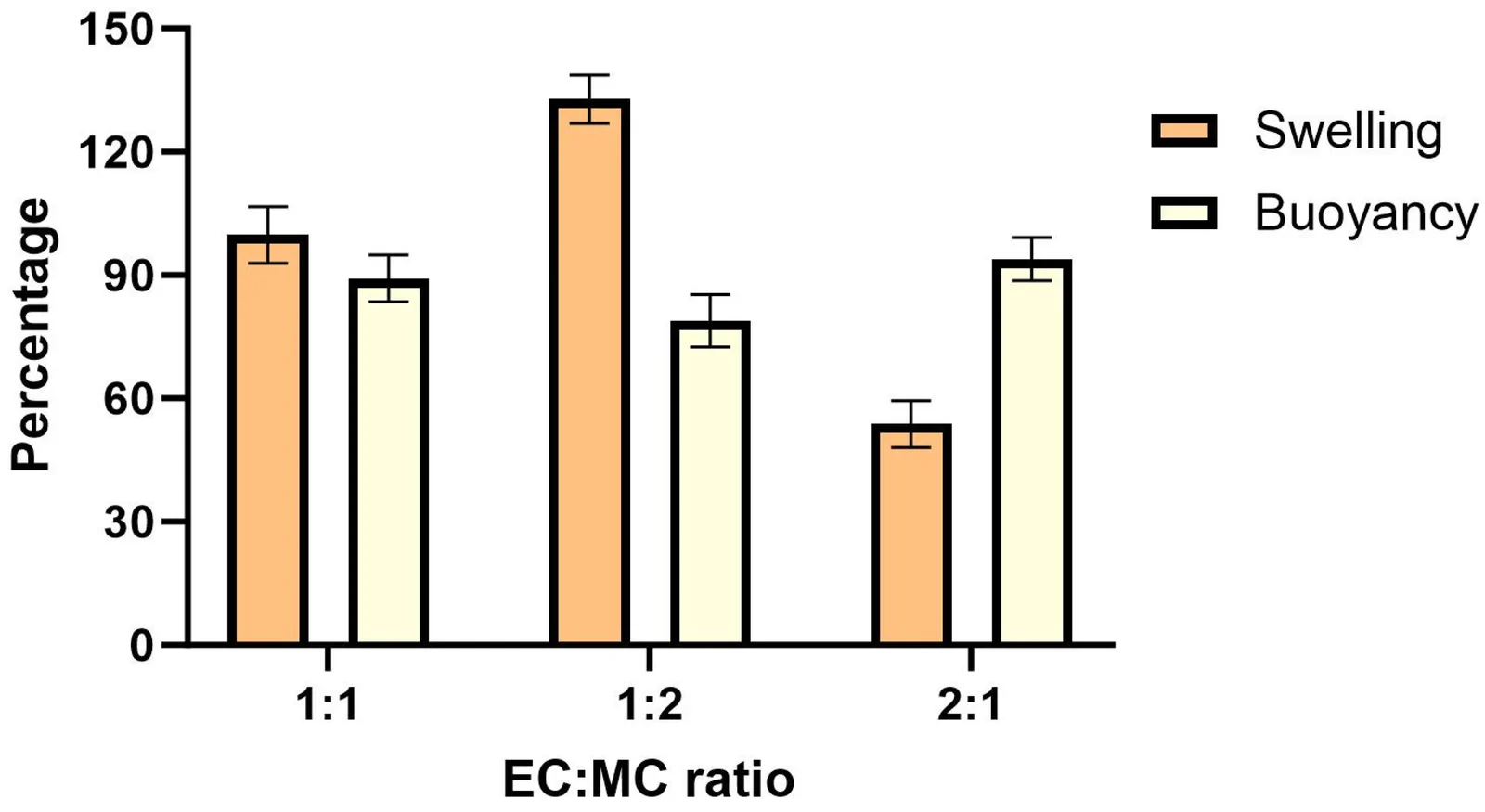
Effect of varying EC: MC ratio on percentage buoyancy and swelling behaviors of TFNs, respectively.

EC exhibited a more pronounced effect on buoyancy (Figure 1). This is likely due to its hydrophobic nature, which results in the formation of matrix with less permeability, thereby enhancing floatation. The formulation with the polymer composition of EC: MC in a 2:1 ratio showed the highest buoyancy (93.90 ± 3.34%), due to hydrophobic nature of EC, and confirms its gastroretentive potential. However, in vitro buoyancy does not clearly demonstrate the gastric retention of intact TFNs in vivo. On the contrary, higher amount of MC reduced the buoyancy and enhanced the swelling. The hydrophilic nature of MC might have favored the creation of additional channels across TFNs with subsequent enhancement of swelling of polymer compared to EC (Figure 1). This change in density resulting from water diffusion might have contributed to subsequent swelling and settling of TNFs [40,43].

### 3.3. ATR-FTIR Spectroscopy

**Figure 2:**
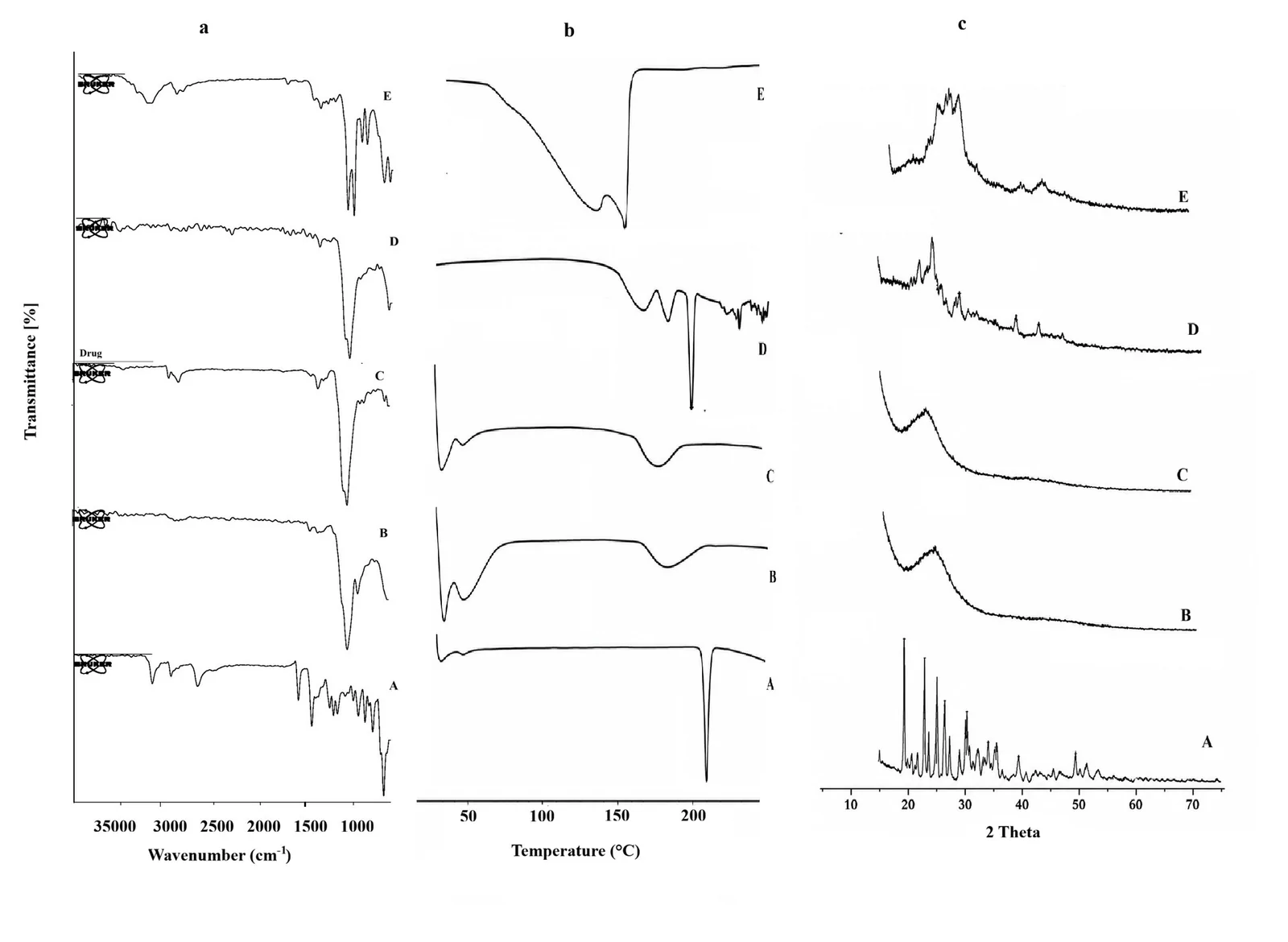
Analytical characterization of (A) pure drug, (B) MC, (C) EC, (D) physical mixture, and (E) optimized TFNs by (**a**) ATR-FTIR spectroscopy, (**b**) DSC and (**c**) PXRD.

ATR-FTIR spectra revealed distinctive peaks characteristic of drug, EC, MC, and optimized formulation (Figure 2a). Spectrum of MC represents broad peak around 3436.91 cm^−1^, indicating O-H stretching vibrations; 2900.53 cm^−1^ C-H stretching vibrations from the methyl and methylene groups; C-H bending vibrations at 1451.81 cm^−1^ and 1370.41 cm^−1^; and C-O-C stretching vibrations at 1052.12 cm^−1^ highlighting ether linkages in methocel structure. Spectrum of EC exhibits broad peak around 3459.42 cm^−1^, reflecting O-H stretching vibrations; 2971.30 cm^−1^ and 2867.38 cm^−1^ are attributed to C-H stretching vibrations from ethyl groups; 1370.04 cm^−1^ corresponds to C-H bending vibrations of the ethyl groups; and 1057.74 cm^−1^ indicates C-O-C stretching vibrations, highlighting ether linkages in the EC structure. The spectrum of tapentadol shows characteristic peaks indicating the presence of amine groups (N-H stretching and bending at 3158.84 cm^−1^ and 1590.84 cm^−1^, respectively), aliphatic hydrocarbons (C-H stretching at 2958.32 cm^−1^), and carbonyl groups (C=O stretching at 1743.20 cm^−1^); C-N stretching at 1254.72 cm^−1^ and 1214.10 cm^−1^; and C-O stretching vibrations at 1171.54 cm^−1^ and 1088.16 cm^−1^, pointing to ether or ester functionalities. The peaks at 1007.71 cm^−1^, 947.58 cm^−1^, 875.13 cm^−1^, 792.80 cm^−1^, and 675.14 cm^−1^ are indicative of aromatic C-H bending vibrations. The major absorption peak of TAP was observed at wavenumber similar to those of pure drug in spectrum of TFNs. However, no prominent new peaks or substantial spectral shifts were observed. The findings suggested that no apparent chemical interaction between formulation components and TAP was observed, confirming successful drug encapsulation in TFNs.

### 3.4. DSC

Thermogram of pure tapentadol exhibited distinct, sharp melting endothermic peak at 208.16 °C, indicating its crystalline behavior. On the contrary, EC and MC exhibited broader melting peaks at approximately 175 °C and 185 °C, respectively. The physical mixture of TAP with EC and MC exhibited the characteristics thermal events of the polymers at approximately 173 °C and 181 °C, along with the characteristic endothermic peak of TAP at 205 °C, indicating no notable interaction between the drug and the polymers. However, thermogram of optimized formulation presented a broad endothermic peak at approximately 150 °C (Figure 2b), while the sharp characteristic melting peak of TAP was no longer evident. The broadening and shifting of thermal events, together with the disappearance of sharp TAP peak, might indicate molecular dispersion of TAP within the polymeric matrix [44,45].

### 3.5. PXRD

The diffractograms of pure drug revealed a series of sharp and intense diffraction peaks at 2θ value of 14.85°, 18.59°, 22.12°, 25.86°, 29.65°, and 44.71° (Figure 2c). These distinct peaks highlight the crystalline nature of the drug, underscoring its well-defined molecular structure. While the diffractograms of Ethocel and Methocel exhibited multiple diffused peaks indicating their amorphous nature, significant sharp peaks corresponding to the pure drug were absent in the diffractogram of optimized preparation; instead, a broad halo was observed.

Collectively, DSC and PXRD results indicated a significant decrease in the long-range crystalline order of TAP in the TFNs, indicative of a predominantly amorphous or highly disordered state of TAP within the polymeric matrix [31,41]. The solid-state transition observed might be contributed by combined effects of polymer-mediated molecular dispersion and encapsulation during the nanoparticle formation, along with rapid solidification during the freeze-drying process. The retention of characteristic TAP FTIR bands in TFN spectra further indicated the transformation of physical state of the drug, with no evidence of any chemical interaction [41].

### 3.6. SEM

**Figure 3:**
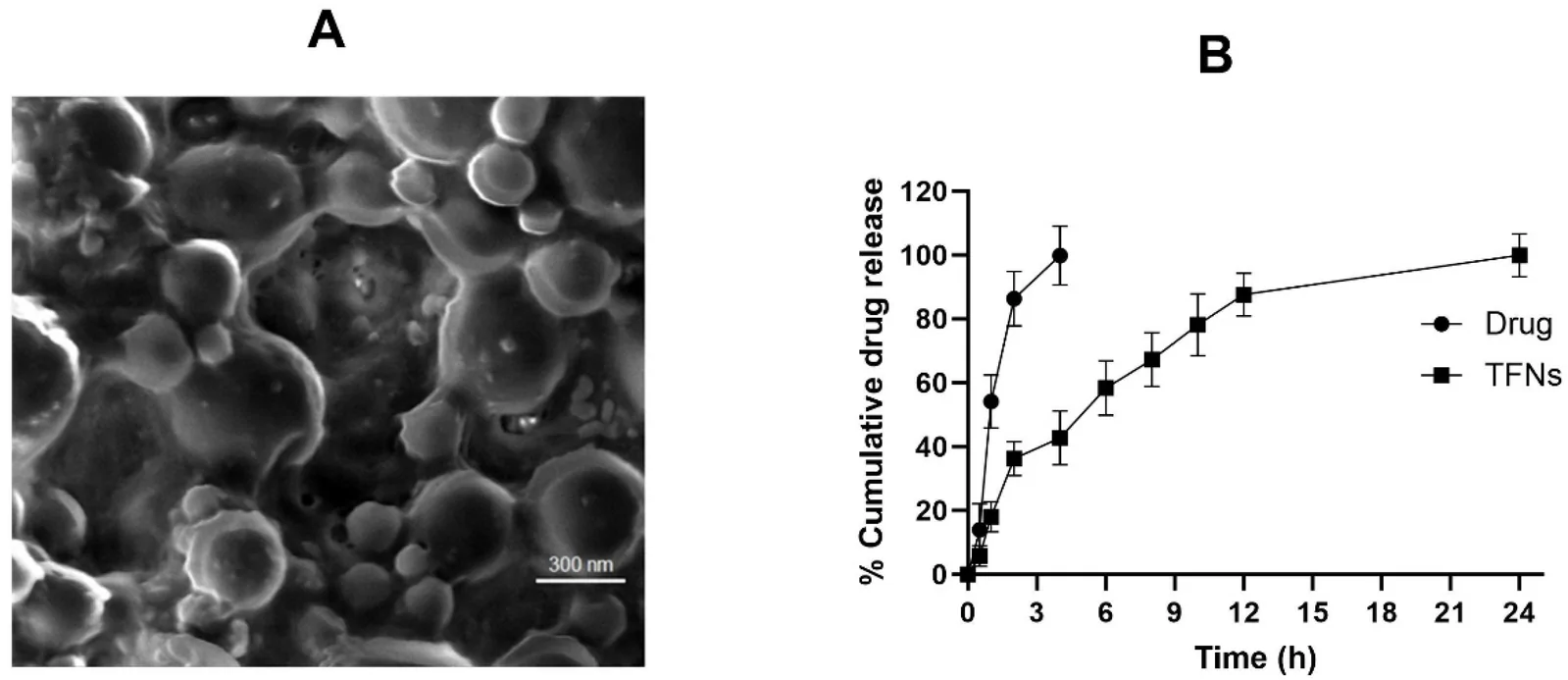
(**A**) SEM images of TFNs. (**B**) In vitro release profile of pure drug and optimized TFNs, respectively.

SEM images revealed that optimized TFNs possessed spherical shape (Figure 3A). However, particles possessed uneven surface with signs of numerous depressions due to solvent evaporation. This morphology of TFNs substantiated controlled drug release with remarkable buoyancy [44]. SEM provides morphological information of dried solid matrix and does not necessarily reflect the dispersion state of the nanoparticles following reconstitution. The removal of water during lyophilization might have allowed the nanoparticles to approach each other and resulted in apparent clusters or fused-looking structures in the dried state.

### 3.7. In Vitro Drug Release

The in vitro release profiles of pure TAP and optimized TFNs conducted in SGF pH 1.2 are represented in Figure 3B. Pure drug showed a rapid release, achieving nearly 100% release within 4 h. On the contrary, optimized TFNs showed sustained drug release extending up to 24 h, due to unique composition of polymer matrix (Figure 3B). EC, being a hydrophobic polymer, acts as a diffusion barrier, whereas hydrophilic nature of MC imparts more porous and loosely packed structure to TFNs, facilitating ease of drug diffusion [45,46]. The significantly faster release rate of free TAP compared to TFNs (*p* < 0.05) suggested that membrane transport was not a major rate limiting factor under experimental conditions. The present study focused mainly on evaluating drug release in simulated gastric conditions to characterize the capacity of the floating TFNs to sustain the drug release in the stomach. However, a sequential dissolution study with initial simulated gastric phase followed by simulated intestinal fluid (SIF) would be a more physiologically representative study of the drug release behavior when gastric-to-intestinal transition occurs. This type of experiment was not carried out in the present study and has now been recognized as a limitation. In a future study, sequential SGF to SIF dissolution testing will be used to assess whether the sustained release behavior of TFNs is maintained following transition to intestinal conditions.

The release data were analyzed using various kinetic models. The regression coefficients (R^2^) obtained for zero-order, first-order, Higuchi, and Korsmeyer–Peppas models were 0.96, 0.78, 0.95, and 0.98, respectively. Higher R^2^ value for the Korsmeyer–Peppas equation and value of release exponent (n = 0.71) suggested anomalous diffusion, implying that drug diffusion, polymer relaxation and dissolution collectively modulated the drug release.

### 3.8. Stability

**Figure 4:**
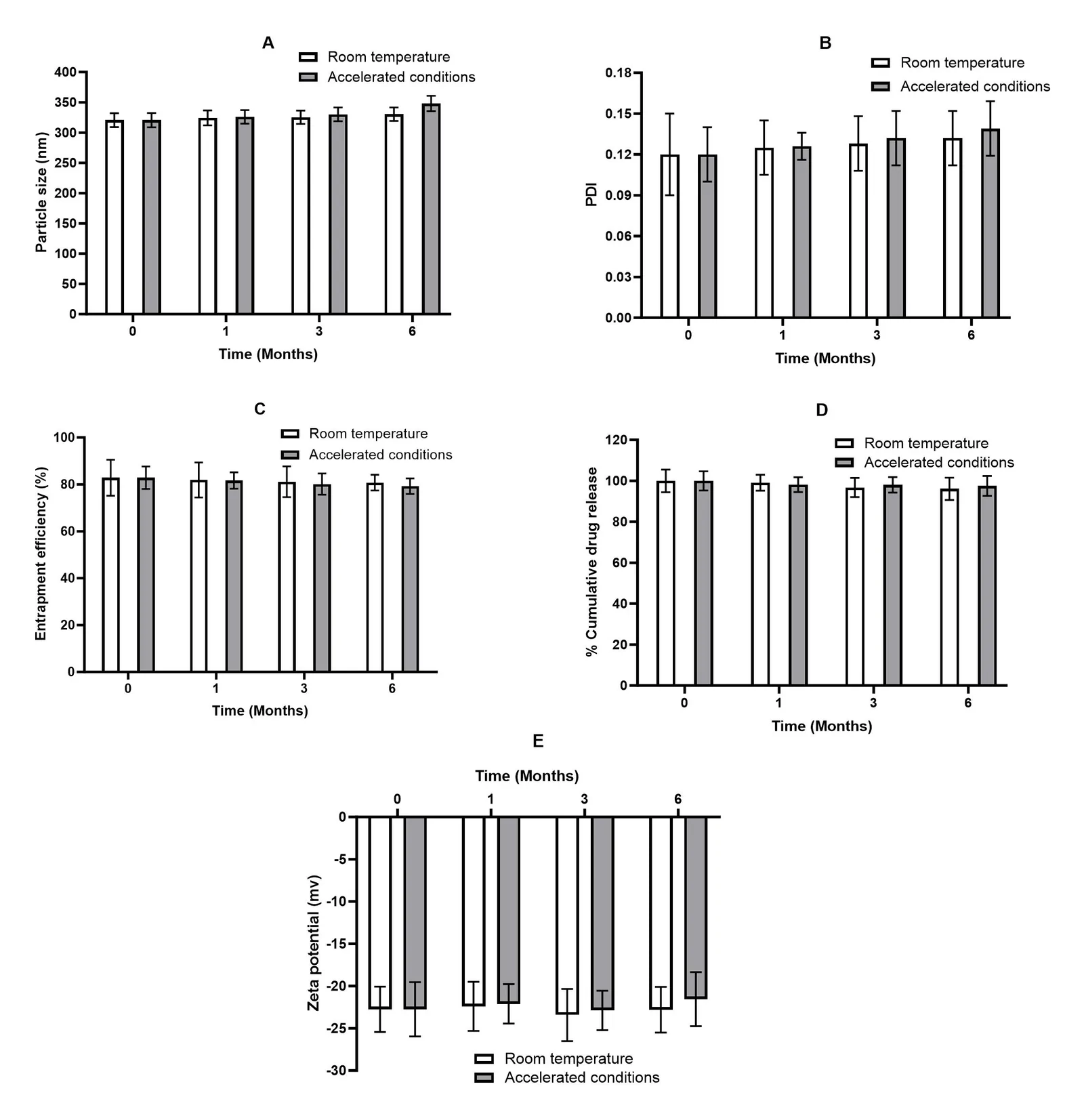
Impacts of different storage conditions on (**A**) average particle size, (**B**) PDI, (**C**) encapsulation efficiency, (**D**) in vitro drug release behavior and (**E**) zeta potential.

The optimized TFNs showed no sign of progressive growth of the nanoparticles, significant aggregation-related increase in PDI, loss of surface charge characteristics, drug leakage, or significant change in the release profile during the storage under both accelerated and room temperature conditions (*p* < 0.05) (Figure 4). Moreover, no significant changes in the visual characteristics of the lyophilized product were seen. The maintained entrapment efficiency and essentially unchanged drug release behavior during the tested time range has provided the functional evidence that no major solid-state change occurred during storage. In addition, no significant change in the particle size or PDI after the reconstitution under the experimental conditions (*p* < 0.05) suggested that the nanoparticulate dispersion characteristics are preserved. However, quantitative redispersibility testing and solid-state characterization by DSC/PXRD before and after storage would give more proof of the lack of irreversible aggregation and drug recrystallization for subsequent pharmaceutical development. These studies will be especially significant during process scale up and validation and development of a clinically suitable lyophilized nanoparticle product.

### 3.9. Pharmacokinetic Profile

**Figure 5:**
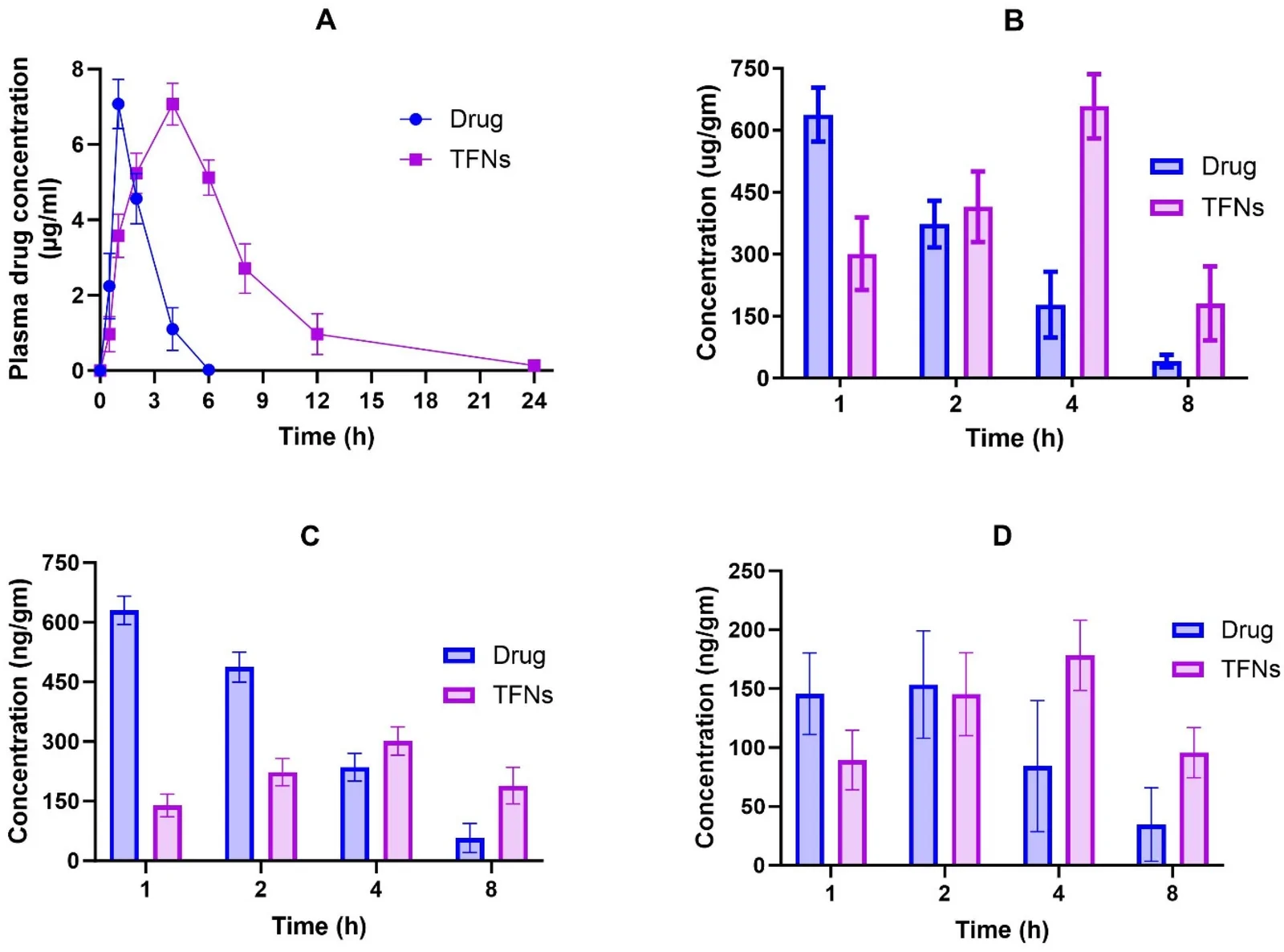
(**A**). Plasma drug concentration–time profiles of tapentadol and optimized TFNs. Bio-distribution of pure drug and optimized TFNs in (**B**) stomach, (**C**) liver, and (**D**) brain.

Oral dosing of plain TAP solution resulted in rapid increase in plasma drug concentration, indicating swift absorption. Cmax of TAP was achieved after 1 h (Figure 5A). In contrast, TFNs demonstrated a more gradual and prolonged absorption profile. No significant differences were observed in Cmax of TFNs (7.06 ± 1.70 µg/mL) and pure TAP, while a significant increase in Tmax (*p* < 0.05) was observed for TFNs, confirming slow and prolonged drug absorption behavior of formulation in vivo. The statistically significant increase in AUC_0–24 h_ by 3.36-fold was observed with TFNs compared to plain TAP (*p* < 0.05) (Figure 5A). This enhancement can be attributed to the prolonged drug release behavior of the nanoparticles, which facilitated sustained drug absorption and increased systemic exposure than plain TAP solution. In addition, TFNs facilitated 1.75-fold increase in mean residence time (MRT) of TAP after oral dosing compared to pure drug, confirming prolonged drug presence in the systemic circulation (Table 2). TFNs led to decrease in elimination rate constant (Ke) and increase in half-life (t_1/2_) which further confirmed prolonged systemic exposure. The increased systemic exposure following TFNs was in agreement of prolonged drug release behavior and buoyancy of TFNs, which might have prolonged gastric drug exposure and retention [11,40]. Thus, sustained drug delivery into systemic circulation from TFNs can help in managing neuropathic pain more effectively, mitigating the concerns pertinent to the short half-life of TAP.

### 3.10. Organ Distribution

Figure 5B–D represents the distribution pattern of tapentadol in stomach, liver and brain at different time intervals after single oral dose administration of pure drug and TFNs. The maximum drug concentration was observed in stomach during the initial period for up to 1 h for pure drug. Pure drug concentration started decreasing significantly after an hour in stomach compared to TFNs and had attained accountable concentration in liver and brain. TFNs exhibited a significantly (*p* < 0.05) higher drug concentration in stomach for longer duration compared with pure tapentadol, indirectly suggesting increased gastric drug exposure and retention. However, without in vivo imaging, it is difficult to conclude whether the intact TFNs in the stomach were buoyant or not for the duration observed. Hepatic tapentadol concentration declined rapidly after administration of free drug. However, time course of hepatic drug concentration achieved with TFNs was clearly different (Figure 5C). Hence, the variation in hepatic exposure levels, along with higher and prolonged systemic tapentadol exposure following TFN administration, might be associated with altered absorption and systemic disposition of tapentadol. Brain tapentadol concentrations declined rapidly after pure drug administration compared to TFNs, which produced a more prolonged brain exposure profile. The prolonged systemic drug availability with TFNs, as evidenced from the results of pharmacokinetic study, might have contributed to sustained brain tapentadol concentrations [45].

### 3.11. Motor Coordination and Balance

**Figure 6:**
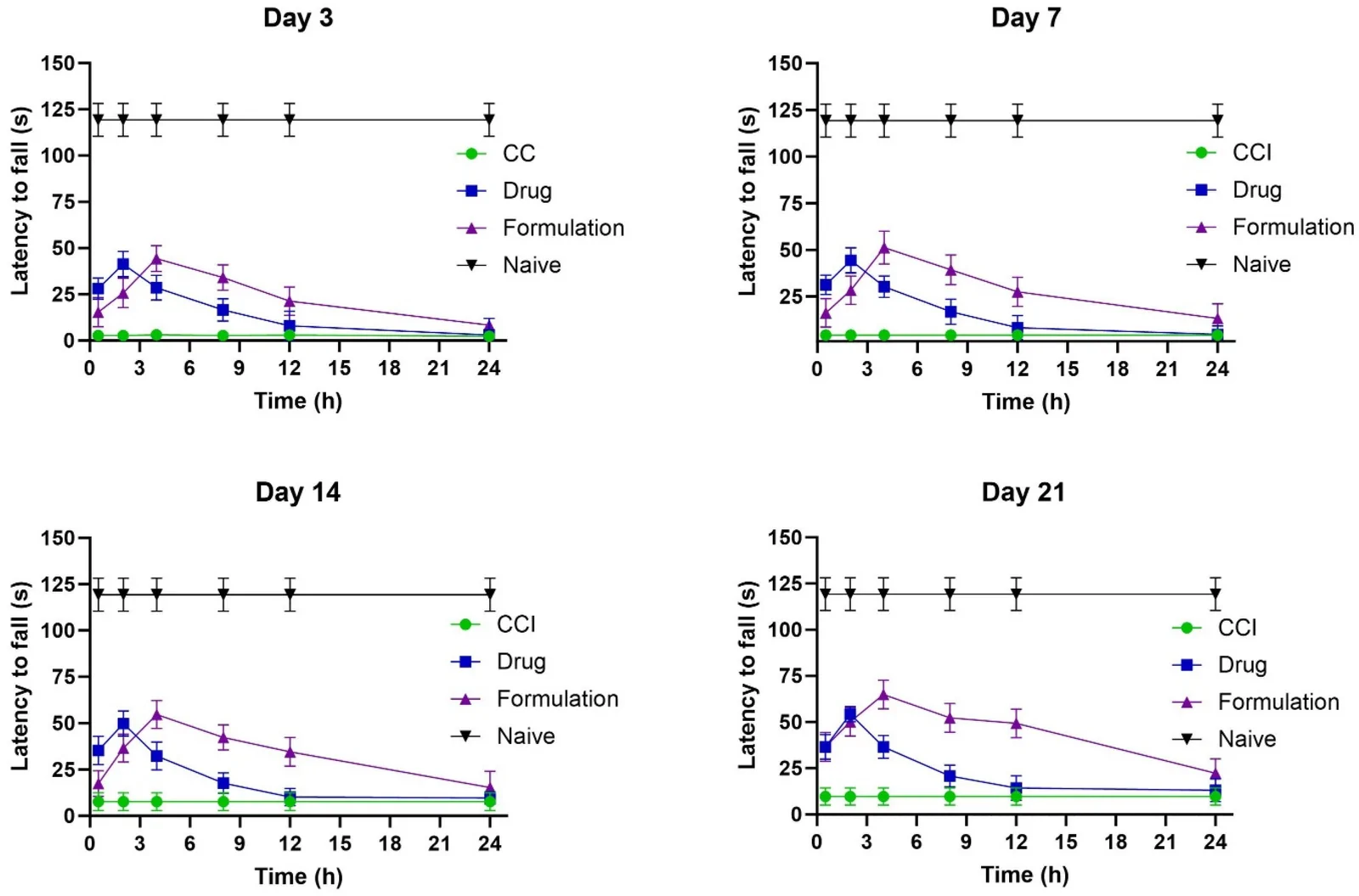
Influences of distinct treatments on motor coordination and balance during Rota rod test on days 3, 7, 14, and 21, respectively.

Motor coordination and balance were assessed by measuring the time the animal remained on the rotating rod before falling. The sham group of animals showed no noticeable change in performance during the entire study period. In contrast, the CCI group exhibited a considerable reduction in the latency to fall, indicating motor impairment caused by nerve injury, which differed significantly from the sham group (*p* < 0.05) (Figure 6) [47]. Treatment with pure drug and TFNs improved the coordination ability of rats, as reflected by increased fall latency compared to vehicle group. Among both treatments, TFNs produced a more stable and long-lasting improvement in motor performance, showing statistically significant differences in comparison with the pure TAP and vehicle groups (*p* < 0.05) (Figure 6).

### 3.12. Mechanical Allodynia

**Figure 7:**
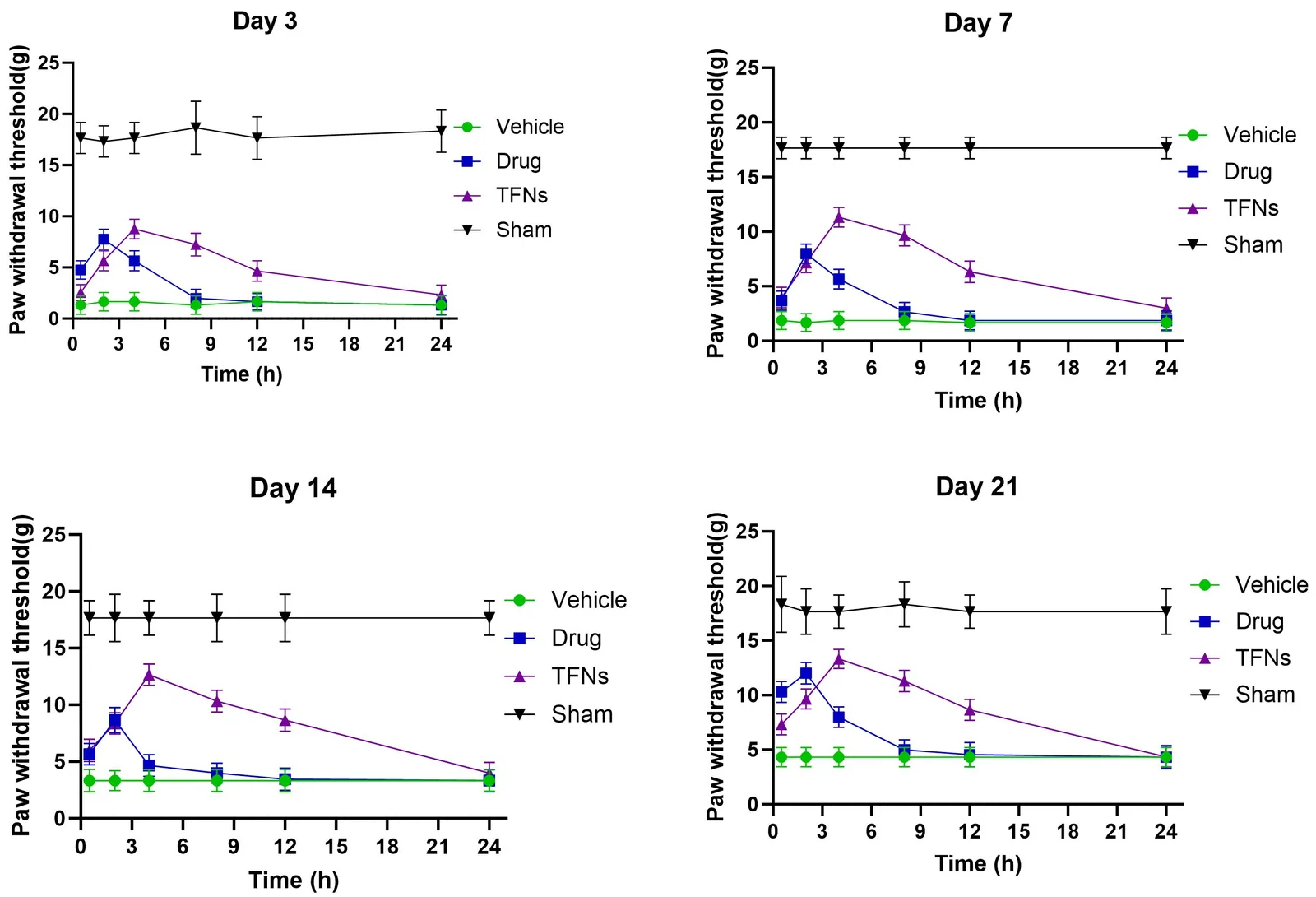
Impacts of distinct treatments on mechanical allodynia on days 3, 7, 14, and 21, respectively.

Animals with CCI exhibited persistent mechanical hypersensitivity of the hind paw to von Frey stimulation throughout the 21-day study period, whereas sham controls maintained stable paw withdrawal thresholds (17.33 ± 1.52 g to 18.66 ± 2.58 g). Oral administration of tapentadol or TFNs remarkably attenuated CCI-induced tactile allodynia (*p* < 0.05), while the vehicle produced no effect (Figure 7). The magnitude and duration of anti-hypersensitivity effects were of longer duration with TFNs and exhibited significant differences from pure drug at relevant observation time points. Notably, TFNs significantly prolonged the anti-allodynic response relative to TAP solution (*p* < 0.05), consistent with sustained systemic and brain tapentadol exposure observed in pharmacokinetic and tissue distribution studies. However, no apparent attenuation of anti-allodynic response over the course of study following repeated daily administration was observed. Tapentadol is a unique novel nociceptive drug for pain relief through an integrated dual mechanism of action, encompassing both µ-opioid receptor-mediated analgesia and norepinephrine reuptake inhibition-mediated enhancement of descending pain inhibitory mechanisms. Moreover, the effects observed with TFNs could be attributed to the combined characteristics of improved gastric drug exposure time and the sustained release of tapentadol [20,40]. Collectively, results highlight TFNs as a promising formulation strategy to extend the therapeutic efficacy of tapentadol and reduce the need for frequent dosing of this short-acting analgesic in neuropathic pain management.

## 4. Conclusions

Tapentadol-loaded nanoparticles were successfully prepared to develop a novel therapeutic approach for the management of neuropathic pain. The formulation utilized matrix-forming polymers that enhanced drug entrapment efficiency and buoyancy, supported gastroretentive potential, and provided a sustained drug release profile. These formulation attributes collectively resulted in improved bioavailability and therapeutic performance of tapentadol. Pharmacodynamic studies showed a significant reduction in neuropathic pain behavior with TFNs, providing a more prolonged anti-allodynia response over the observation period. The present work establishes a relationship among formulation, biopharmaceutical, and pharmacodynamic properties, instead of merely developing another sustained-release dosage form. Nevertheless, further studies focusing on large-scale production, long-term safety, and clinical evaluation are necessary before the formulation can be advanced for clinical application.

## Figures and Tables

**Table 1:** Optimization of different formulation variables depending on colloidal properties and entrapment efficiency of TFNs.

Formulation Code	Polymer Ratio (EC: MC)	Drug: Polymer Ratio	Surfactant (%*w*/*v*)		Sonication Time (min)		External Phase Volume (mL)	Particle Size (nm ± SD)	Zeta Potential (mV ± SD)	Polydispersity Index (PDI ± SD)	Entrapment Efficiency (%± SD)
			Span −80	Tween −80	Pre	Post					
G1	2:1	1:4	0.5	-	10.5	12.5	30	740.66 ± 53.68	−21.73 ± 3.15	0.67 ± 0.02	61.10 ± 5.17
G2	1:1	1:4	0.5	-	10.5	12.5	30	320.66 ± 17.50	−22.75 ± 2.68	0.12 ± 0.02	82.85 ± 6.57
G3	1:2	1:4	0.5	-	10.5	12.5	30	501.06 ± 25.85	−24.86 ± 3.80	0.55 ± 0.02	70.55± 5.45
G4	1:1	1:8	0.5	-	10.5	12.5	30	301.34 ± 15.8	−11.55 ± 22.66	0.22 ± 0.02	41.98 ± 8.18
G5	1:1	1:2.67	0.5	-	10.5	12.5	30	359.36 ± 22.36	−17.56 ± 2.56	0.25 ± 0.02	74.23 ± 5.69
G6	1:1	1:4	0.5	-	7.5	12.5	30	1141.23 ± 47.17	−12.73 ± 4.37	0.95 ± 0.04	42.67 ± 5.23
G7	1:1	1:4	0.5	-	10.5	10.5	30	538.36 ± 28.29	−16.14 ± 2.98	0.64 ± 0.02	65.97 ± 5.22
G8	1:1	1:4	0.5	-	12.5	12.5	30	454.33 ± 12.50	−17.16 ± 8.15	0.80 ± 0.05	42.82 ± 5.46
G9	1:1	1:4	0.5	-	10.5	15	30	919.33 ± 29.36	−9.63 ± 5.13	0.65 ± 0.02	35.60 ± 4.24
G10	1:1	1:4	0.5	-	10.5	12.5	20	206.33 ± 12.13	−14.53 ± 2.51	0.26 ± 0.07	67.75 ± 4.47
G11	1:1	1:4	0.5	-	10.5	12.5	40	662.66 ± 49.16	−19.96 ± 3.41	0.93 ± 0.04	42.10 ± 4.23
G12	1:1	1:4	-	0.5	10.5	12.5	30	403.33 ± 25.17	−14.61 ± 2.66	0.15 ± 0.06	67.50 ± 5.38
G13	1:1	1:4	1	-	10.5	12.5	30	299.23 ± 16.73	−17.71 ± 4.78	0.11 ± 0.05	60.65 ± 5.78

**Table 2:** Comparative pharmacokinetic parameters of tapentadol and optimized TFNs.

Parameters	Pure Drug	Optimized TFNs
Cmax (µg mL^−1^)	7.07 ± 1.04	7.06 ± 1.7
Tmax (h)	1.0 ± 0.12	4.0 ± 0.11
Ke (h^−1^)	0.63 ± 0.15	0.20 ± 0.08
t_1/2_ (h)	1.09 ± 0.12	3.48 ± 0.34
MRT (h)	1.42 ± 0.23	2.49 ± 1.25
AUC (µg h mL^−1^)	15.51 ± 1.37	52.08 ± 3.77

## Data Availability

The data underlying this study are available in the published article.

## Abbreviations

The following abbreviations are used in this manuscript:

ATR-FTIR	Attenuated total reflectance–Fourier Transform Infrared Spectroscopy
CCI	Chronic constriction injury
DSC	Differential scanning calorimetry
EC	Ethocel
HPMC	Hydroxypropyl methylcellulose
MC	Methocel
NSAIDS	Non-steroidal anti-inflammatory drugs
PVA	Polyvinyl alcohol
RH	Relative humidity
RP-HPLC	Reversed-phase high-performance liquid chromatography
SEM	Scanning electron microscopy
SGF	Simulated gastric fluid
TAP	Tapentadol
TFNs	Tapentadol-loaded floating nanoparticles
XRD	X–ray diffraction
